# Neural Correlates of Fall Risk in People Aged 50 and Over With Chronic Low Back Pain: A Multimodal MRI Study

**DOI:** 10.1002/ejp.70380

**Published:** 2026-09-11

**Authors:** Mahsa Seydi, Kim Delbaere, Meghan Ambrens, James K. Richardson, Caroline D. Rae, Jun Cao, Perminder S. Sachdev, Louise Lavrencic, Michael A. Green, Kimberley S. van Schooten

**Affiliations:** ^1^ School of Population Health University of New South Wales Kensington New South Wales Australia; ^2^ Falls, Balance and Injury Research Centre Neuroscience Research Australia Sydney New South Wales Australia; ^3^ School of Health Sciences University of New South Wales Kensington New South Wales Australia; ^4^ Department of Physical Medicine and Rehabilitation University of Michigan Ann Arbor Michigan USA; ^5^ School of Psychology University of New South Wales Kensington New South Wales Australia; ^6^ Centre for Healthy Brain Ageing (CHeBA), School of Clinical Medicine, Faculty of Medicine and Health University of New South Wales Sydney New South Wales Australia; ^7^ Neuropsychiatric Institute The Prince of Wales Hospital Sydney New South Wales Australia; ^8^ Aboriginal Health & Ageing Program, Neuroscience Research Australia Randwick New South Wales Australia; ^9^ School of Clinical Medicine University of New South Wales Kensington New South Wales Australia

**Keywords:** chronic low back pain, cognitive‐motor integration, executive function, fall risk, gait quality, multimodal MRI

## Abstract

**Background:**

Chronic low back pain (CLBP) is common in older people and contributes to disability, physical inactivity, cognitive deficits and neural alterations that may increase fall risk. We examined associations between multimodal brain measures and daily‐life gait quality and other fall risk factors in older people with CLBP.

**Methods:**

Thirty‐three adults aged ≥ 50 years with CLBP completed assessments of physical function, physical activity, and executive function. Daily‐life gait quality was assessed using a waist‐worn accelerometer. Neuroimaging included diffusion MRI, T1‐weighted MRI and magnetic resonance electrical properties tomography.

**Results:**

Gait quality was associated with tissue conductivity in bilateral cerebellar white matter, pallidum and thalamus, but not with structural measures. Short Physical Performance Battery (SPPB) score was associated with fibre density in visual and thalamo‐occipital tracts, and hippocampal, thalamic and putamen volumes. Walking duration and daily‐life gait speed were associated with cerebellar and subcortical volumes and frontal–parietal conductivity. Physical activity was associated with hippocampal, cerebellar, parietal, and frontal conductivity. Inhibitory control was associated with sensorimotor fibre density and medial orbitofrontal conductivity.

**Conclusions:**

In older people with CLBP, gait, physical function and executive function are supported by complementary neural networks, with tissue conductivity providing additional insight into real‐world gait performance and physical function.

**Significance Statement:**

This study highlights brain mechanisms underlying fall risk in older people with chronic low back pain. Tissue conductivity in cerebellar, subcortical and orbitofrontal regions was linked to balance, gait quality and inhibitory control, providing insights beyond structural MRI. Findings suggest that alterations across neural circuits involved in motor control, cognitive processing and physical activity may contribute to mobility decline. Understanding these neural pathways may help guide targeted interventions to improve balance and reduce fall risk in people with chronic low back pain.

## 1 Introduction

Chronic low back pain (CLBP) is a leading cause of years lived with disability worldwide, with prevalence increasing significantly with age (Ferreira 2023). In Australia, one in six adults reports back problems, contributing to an estimated $3.4 billion in healthcare costs in 2019–20 (Australian Institute of Health and Welfare [AIHW] 2024). When pain becomes chronic, persisting for more than 12 weeks, it is associated with greater disability, reduced quality of life, and high healthcare utilisation (Geneen et al. 2017; Kovacs et al. 2005). Importantly, chronic pain in older adults has been consistently associated with increased risk of falls, impaired balance, and fear of falling (Patel et al. 2014). Large population‐based and longitudinal studies have reported higher fall rates and poorer balance performance among older adults with persistent pain, including low back pain (Marshall et al. 2016; Patel et al. 2014; Welsh et al. 2019). Despite this established association, the underlying neural and behavioural mechanisms linking CLBP to mobility impairment, particularly balance deficits and falls, remain poorly understood.

People living with CLBP frequently exhibit reduced physical activity levels, which, in combination with ongoing pain, can lead to structural and functional brain changes (Parker et al. 2017; Yang and Chang 2019). These alterations extend beyond pain‐processing regions to regions critical for balance and motor control and may contribute to impaired mobility and increased risk of falls (Hicks et al. 2020; Hupfeld et al. 2022). Evidence suggests these deficits are not solely explained by musculoskeletal or sensory impairments, but also reflect broader disruptions in cognitive and neurological function (Zhang et al. 2019). Cognitive domains such as executive function, attention, and processing speed are increasingly recognised as key contributors to balance and falls (Hsu et al. 2012). Executive dysfunction may reduce adaptability to environmental challenges, while impaired attention and processing speed can compromise postural control, delay reaction times and hinder sensory integration, all of which elevate fall risk (Forte et al. 2019; Sturnieks et al. 2025).

Neuroimaging studies have identified multiple structures implicated in balance, including the cerebellum, basal ganglia, thalamus, hippocampus, parietal cortex and frontal regions such as the inferior orbitofrontal cortex, motor cortex and supplementary motor area (SMA) (Surgent et al. 2019). These same regions are engaged in pain processing, suggesting overlapping neural pathways through which CLBP may impact balance (Bingel et al. 2002). Functional MRI (fMRI) studies suggest altered functional connectivity during motor imagery in CLBP, consistent with disrupted motor planning and sensorimotor integration (Lamichhane et al. 2021; Vrana et al. 2015). Structural MRI (sMRI) studies suggested volumetric changes in pain‐related and motor regions, while diffusion MRI (dMRI) studies demonstrated reduced white matter integrity, associated with impaired proprioception and connectivity (Henn et al. 2023; Zhai et al. 2020). Despite these findings, few studies have examined how brain integrity in CLBP relate directly to daily‐life balance performance or to established fall risk factors such as balance and executive function. A small number of studies have investigated balance performance and fall‐related outcomes in people with CLBP, demonstrating impairments in postural control, altered gait strategies and increased fear of falling compared to healthy controls (Brumagne et al. 2008; Ruhe et al. 2011; Suri et al. 2022). In parallel, neuroimaging research has identified structural and functional alterations in regions involved in both pain processing and motor control in CLBP, including reduced grey matter volume, altered functional connectivity and compromised white matter integrity (Apkarian et al. 2004; Henn et al. 2023; Yang et al. 2023). Yet none of the neuroimaging work has been linked to ecologically valid measures of fall risk, leaving the neural basis of real‐world mobility impairment in CLBP poorly understood.

To address this gap, this study investigated the neural correlates of balance in people with CLBP, focusing on daily‐life gait quality, a sensitive and ecologically valid marker of real‐world balance and fall risk (van Schooten, Pijnappels, et al. 2019). The primary aim of this study was to examine the associations between multimodal brain measures (i.e., white matter microstructure, grey matter volume, tissue conductivity) and daily‐life gait quality. We also explored associations with other fall risk factors, including physical and executive function. By combining advanced neuroimaging techniques with detailed cognitive and physical assessments, this study addresses a critical gap in understanding how chronic pain‐related brain changes contribute to balance impairment and fall risk.

## 2 Methods

### 2.1 Study Design

This cross‐sectional study was conducted as a sub‐study of a pilot randomised controlled trial, the results of which are not yet published but prospectively registered with the Australian New Zealand Clinical Trials Registry (ACTRN12623001266651). The parent trial developed and tested a digital physical activity intervention and an online psychological program in adults aged ≥ 50 years with chronic low back pain, recruiting generally healthy, community‐dwelling adults able to walk independently through the Neuroscience Research Australia (NeuRA) volunteer registry. All participants without contraindications for MRI were eligible and invited to undergo brain imaging at baseline. Of the 40 participants enrolled in the parent trial, 33 (83%) had no MRI contraindications and consented to scanning.

### 2.2 Inclusion Criteria and Screening

Participants were eligible if they were aged 50 years or older, reported having chronic low back pain for at least 3 months, as confirmed using self‐report during screening. Participants were required to live independently in the community, be able to ambulate at least 100–300 m (with or without a walking aid) and communicate effectively in English. Key exclusion criteria included current engagement in guideline‐level physical activity, major cognitive impairment (as measured with a Short Portable Mental Status Questionnaire score of < 8), diagnosed with a medical condition(s) precluding safe participation in physical activity, or contraindications for MRI. In addition, psychological screening was conducted using the Patient Health Questionnaire‐9 (PHQ‐9). Participants scoring ≥ 15 or endorsing suicidal ideation were referred to the eCentreClinic for a clinical psychological risk assessment by a qualified mental health professional. Ethical approval was obtained from the UNSW Human Research Ethics Committee, and all participants provided written informed consent using a secure online REDCap form, which was confirmed verbally at the assessment visit.

### 2.3 Outcome Measures

The primary outcome was daily‐life gait quality, assessed using the McRoberts MoveMonitor (McRoberts). This outcome was selected a priori as the main indicator of real‐world balance and fall risk (van Schooten, Pijnappels, et al. 2019). Secondary outcomes included measures of physical function, including the Short Physical Performance Battery (SPPB), daily‐life walking speed and the Incidental and Planned Exercise Questionnaire for Older People (IPEQ‐W), and executive function, including ReacStick and the Trail Making Test (TMT) (Delbaere et al. 2010; Eckner et al. 2012; Tischler and Petermann 2010; Treacy and Hassett 2018). Multimodal neuroimaging measures included white matter microstructure, grey matter volume and tissue conductivity.

### 2.4 Daily‐Life Gait Quality

Daily‐life gait quality was assessed using the McRoberts MoveMonitor (McRoberts B.V., The Netherlands), a validated waist‐worn activity monitor worn continuously for 7 days prior to their neuroimaging appointment at NeuRA. The device records trunk accelerations during daily activities, periods of walking were identified and a gait quality composite score that is highly predictive of falls was determined (van Schooten, Pijnappels, et al. 2019). This composite score was derived from median weekly values of a weighted sum of autocorrelation at stride frequency, power at step frequency, root mean square of the accelerations and index of harmonicity (Van Schooten et al. 2016). Similar methods were used to estimate habitual daily‐life walking speed (Van Schooten et al. 2016).

### 2.5 Physical Function

#### 2.5.1 IPEQ‐W

Physical activity levels were assessed using the Incidental and Planned Exercise Questionnaire for Older People IPEQ‐W, a validated self‐report tool designed specifically for this population (Delbaere et al. 2010). The questionnaire captures both the frequency and duration of a range of planned physical activities (e.g., structured exercise classes, walking for exercise and home‐based workouts) and incidental activities (e.g., household chores, gardening and walking for transport), providing a comprehensive estimate of weekly physical activity engagement. Total weekly physical activity is calculated in hours per week, with higher values indicating greater activity levels.

#### 2.5.2 Short Physical Performance Battery (SPPB)

SPPB is a widely used assessment of lower‐extremity function in older people (Guralnik et al. 1994). It provides a composite score based on three key components: usual walking speed, balance during progressively challenging standing positions and the ability to rise from a chair. Together, these measures offer a validated indicator of physical performance and functional mobility. The SPPB provides a composite score ranging from 0 to 12, with higher scores indicating better lower‐extremity function.

### 2.6 Executive Function

#### 2.6.1 ReacStick

The ReacStick is a rod‐shaped handheld device used to assess rapid response inhibition and processing speed through a ‘ruler drop’ light‐cued go/no‐go paradigm. Participants were instructed to catch the device when released by the examiner if LED lights on the device illuminated (‘go’ trial) and to inhibit catching when the lights remained off (‘no‐go’ trial). OFF Accuracy is the percentage of trials appropriately not caught when the lights did not illuminate, offering an objective, time‐sensitive (390 ms) measure of inhibitory executive function that is minimally influenced by education or fine motor skills (Lane et al. 2026; van Schooten, Duran, et al. 2019). Higher values of OFF Accuracy indicate better inhibitory control.

#### 2.6.2 Trail Making Test (TMT)

The TMT is a widely used neuropsychological assessment of processing speed, attention and executive function. It includes two parts: TMT‐A, which involves connecting 25 numbered circles in sequence, and TMT‐B, which requires alternating between numbers and letters (e.g., 1‐A‐2‐B). The primary outcome is completion time for each part, with longer durations indicating poorer performance (Tombaugh 2004).

### 2.7 Neuroimaging and Brain Measures

All data were acquired on a Philips Ingenia CX 3T MRI scanner using a 32‐channel head coil. Scans included:

#### 2.7.1 T1‐Weighted MRI

A multishot 3D non‐selective turbo field echo sequence was acquired (TR/TE = 7.9/3.8 ms, voxel size = 0.9 mm^3^, compressed SENSE factor = 2.5). Volumetric segmentation and cortical parcellation were performed using FreeSurfer (2026) (version 7.3.2), applyin g the Desikan–Killiany atlas for cortical regions and probabilistic labelling for subcortical structures (Velázquez et al. 2021). Total intracranial volume (tICV) was automatically estimated by FreeSurfer and included as a covariate in volumetric regression analyses to account for inter‐individual differences in brain size.

#### 2.7.2 Diffusion MRI

Datasets were acquired using a high angular resolution diffusion imaging (HARDI) protocol, optimised for advanced white matter analysis (multishell, single‐shot spin‐echo EPI; multiband factor = 3; SENSE = 1.4; voxel size = 2.4 mm^3^; TR/TE = 3501/80 ms; b‐values = 1000 and 3000 s/mm^2^; 97 gradient directions). Preprocessing and FBA were conducted using MRtrix3 (Tournier et al. 2019). Preprocessing included denoising, correction for eddy currents and motion, bias field correction, and up‐sampling to enhance anatomical contrast and registration. Fibre orientation distributions (FODs) were estimated and nonlinearly registered to a study‐specific template (Raffelt et al. 2011). Fixel‐based metrics included fibre density (FD), fibre cross‐section (FC), and combined fibre density and cross‐section (FDC). Whole‐brain tractography was performed using 20 million streamlines generated from the study template and reduced to 2 million using spherical‐deconvolution informed filtering (SIFT) to reduce reconstruction bias (Smith et al. 2015).

#### 2.7.3 Magnetic Resonance Electrical Properties Tomography (MREPT)

MREPT is an emerging non‐invasive MRI technique that reconstructs tissue conductivity and permittivity, offering biophysical estimates of current propagation that may reflect microstructural properties such as myelin, cellular composition and water distribution, as well as early neurodegenerative or inflammatory changes (Faulkner et al. 2024; Katscher et al. 2009; Liu et al. 2017). Conductivity maps were obtained using a balanced fast field echo sequence (TR/TE 2.55/1.27 ms, flip angle 25°, voxel size 1 mm^3^, FOV 240 × 240 mm^2^) and corresponding phase data, with RF shimming calibrated by 2D DREAM‐based B1+ mapping (Cao et al. 2023). Phase‐based MREPT was applied (*σ* ≈∇^2^φ±/2 μ₀ω), and T1‐weighted images were co‐registered to segment white matter, grey matter and CSF (FSL) to reduce boundary artefacts. Within each tissue, average parabolic fitting of phase data minimised Laplacian‐related noise (Cao et al. 2023; Katscher et al. 2012). Mean regional conductivity values were extracted from FreeSurfer‐derived Desikan–Killiany ROIs.

Age, sex and education were included as covariates in all imaging analyses. Total intracranial volume (tICV) was estimated using SynthSeg 2.0, a deep learning–based segmentation tool for robust brain volumetry, and included as a covariate in all volumetric regression analyses to account for inter‐individual differences in brain size (FreeSurfer 2026).

### 2.8 Statistical Analysis

The primary hypothesis examined the association between brain integrity and daily‐life gait quality, defined a priori as the primary outcome. This was assessed across three complementary imaging modalities (diffusion MRI, grey matter volume and tissue conductivity), reflecting different aspects of brain integrity.

For diffusion MRI analyses, family‐wise error (FWE) correction was applied using connectivity‐based fixel enhancement (CFE), with 5000 permutations testing and a family‐wise error (FWE) of 0.05, given the high dimensionality of these data (Raffelt et al. 2015). For other imaging modalities, no formal correction across modalities was applied; results are interpreted with caution, with emphasis on effect sizes and consistency across modalities rather than isolated statistical significance.

Analyses involving additional behavioural measures (physical and executive function) were pre‐specified as secondary and considered exploratory.

Descriptive statistics were computed for all participant characteristics and outcome measures. Missing data were addressed using Multiple Imputation by Chained Equations (MICE) in R (version 4.2.3) using the mice package (van Buuren and Groothuis‐Oudshoorn 2011). Linear regression models were used to examine associations between brain metrics (grey matter volume, white matter integrity and tissue conductivity) and daily‐life gait quality composite score, physical function (SPPB), physical activity (IPEQ‐W) and executive function measures (ReacStick OFF Accuracy and Trail Making Test performance). FBAs were performed controlling for age, sex, education and tICV (where relevant).

## 3 Results

### 3.1 Participant Characteristics at Baseline

Thirty‐three participants with CLBP enrolled in the *Walking With(out) Pain* study participated in this sub‐study and underwent MRI scanning. Table 1 presents the baseline characteristics. The mean age of participants was 66 years (SD = 10.2), and 85% were female. Participants had an average of 17.6 years of education. Physical function was indicated by a mean SPPB score of 10.7 (SD = 1.4). On average, participants walked for 1 h (SD = 0.5) per day and engaged in 4.7 h (SD = 3) of planned exercise per week. The majority reported mild (40%) or moderate (42%) pain, with only 18% reporting severe pain, based on a self‐reported item assessing overall bodily pain in the past 30 days (response options: mild, moderate or severe). Reaction time and task accuracy metrics were also measured, with ON accuracy averaging 0.85 (SD = 0.11) and OFF accuracy at 0.37 (SD = 0.24).

**TABLE 1:** Baseline characteristics (*n* = 33).

Variable	Mean (SD) or *n* (%)
Age (years)	66 (10.2)
Gender (% female)	28 (85%)
BMI (kg/m^2^)	28.7 (5.1)
Years of education	17.6 (4.1)
Daily life gait quality (*z*‐score)	0.40 (0.88)
SPPB score (0–12)	10.7 (1.4)
Walking duration (h/day)	1.0 (0.5)
Planned exercise (h/week)	4.7 (3)
Total activity (h/week)	27.7 (14)
Trail making test B–A (s)	37.8 (17.5)
Mini‐ACE score (0–30)	27.6 (1.8)
Reaction time (ms)	212.7 (22.6)
ON accuracy (0–1)	0.85 (0.11)
OFF accuracy (0–1)	0.37 (0.24)

### 3.2 Associations With Daily Life Gait Quality

No significant associations were observed between daily‐life gait quality and grey matter volume in any cortical or subcortical regions. Similarly, white matter properties were assessed using FBA metrics, which provide measures of FD, FC and their FDC, and did not show any significant relationships with gait quality.

In contrast, tissue conductivity analyses revealed significant positive associations with gait quality in several subcortical and cerebellar regions. Specifically, higher conductivity in bilateral cerebellar white matter was strongly associated with better gait quality (left: *β* = +38.8 ± 13.9, *p* = 0.0098; right: *β* = +41.6 ± 12.1, *p* = 0.0019). Additionally, increased conductivity in the left and right pallidum (*β* = +27.7, *p* = 0.0288; *β* = +28.6, *p* = 0.0164, respectively) and right thalamus (*β* = +17.8 ± 6.34, *p* = 0.0093) were associated with higher gait quality scores (Table 2).

**TABLE 2:** Associations between regional brain tissue conductivity and daily‐life gait quality.

Outcome	Region	*β* ± SD	*p*
Gait quality	L cerebellum WM	38.8 ± 79.8	0.0098
	R cerebellum WM	41.6 ± 69.4	0.0019
	L pallidum	27.7 ± 68.9	0.0288
	R pallidum	28.6 ± 64.4	0.0164
	R thalamus	17.8 ± 36.4	0.0093

### 3.3 Associations With Physical Function

Neuroimaging markers across all three modalities, grey matter volume, white matter fibre density and tissue conductivity, were significantly associated with physical function outcomes, including daily‐life walking speed, SPPB performance, walking duration and physical activity patterns, with higher brain integrity measures generally associated with better physical function outcomes.

Grey matter volumes showed strong associations with both SPPB scores and daily walking duration. Higher volumes in the right hippocampus, right thalamus, right putamen and bilateral cerebral white matter were significantly associated with better SPPB performance (*p* < 0.018), while smaller lateral ventricle volumes suggested reduced atrophy in individuals with higher SPPB scores. For walking duration, greater volumes in the bilateral thalami, brainstem, cerebellar regions, left ventral diencephalon and right pallidum were associated with longer ambulatory activity, while greater CSF volume was inversely related, indicating global brain preservation may support higher levels of mobility (Table 3).

**TABLE 3:** Grey matter volume regions significantly associated with physical function.

Outcome	Region	Estimate ± SD^a^	*p*
Walking duration	Right thalamus	0.000624 ± 0.000890	0.00042
	Left thalamus	0.000480 ± 0.000890	0.00461
	CSF	−0.0000101 ± 0.0000195	0.00605
	Left cerebellum white matter	0.000178 ± 0.000343	0.00615
	Right pallidum	0.001721 ± 0.00347	0.00823
	Left ventral DC	0.000841 ± 0.00183	0.01377
	Brainstem	0.000140 ± 0.000308	0.01437
	Right cerebellum cortex	0.0000617 ± 0.000140	0.01725
SPPB	Right hippocampus	0.001467 ± 0.00264	0.00354
	Right thalamus	0.001295 ± 0.00247	0.00554
	Right cerebral white matter	0.0000581 ± 0.000114	0.00699
	Right putamen	0.001809 ± 0.00361	0.00773
	Right lateral ventricle	−0.0000619 ± 0.000140	0.01710
	Left cerebral white matter	0.0000539 ± 0.000122	0.01756
	Left lateral ventricle	−0.0000582 ± 0.000132	0.01776

^a^ Associations adjusted for tICV, age, sex and education. Only regions with *p* < 0.05 are shown.

**FIGURE 1:**
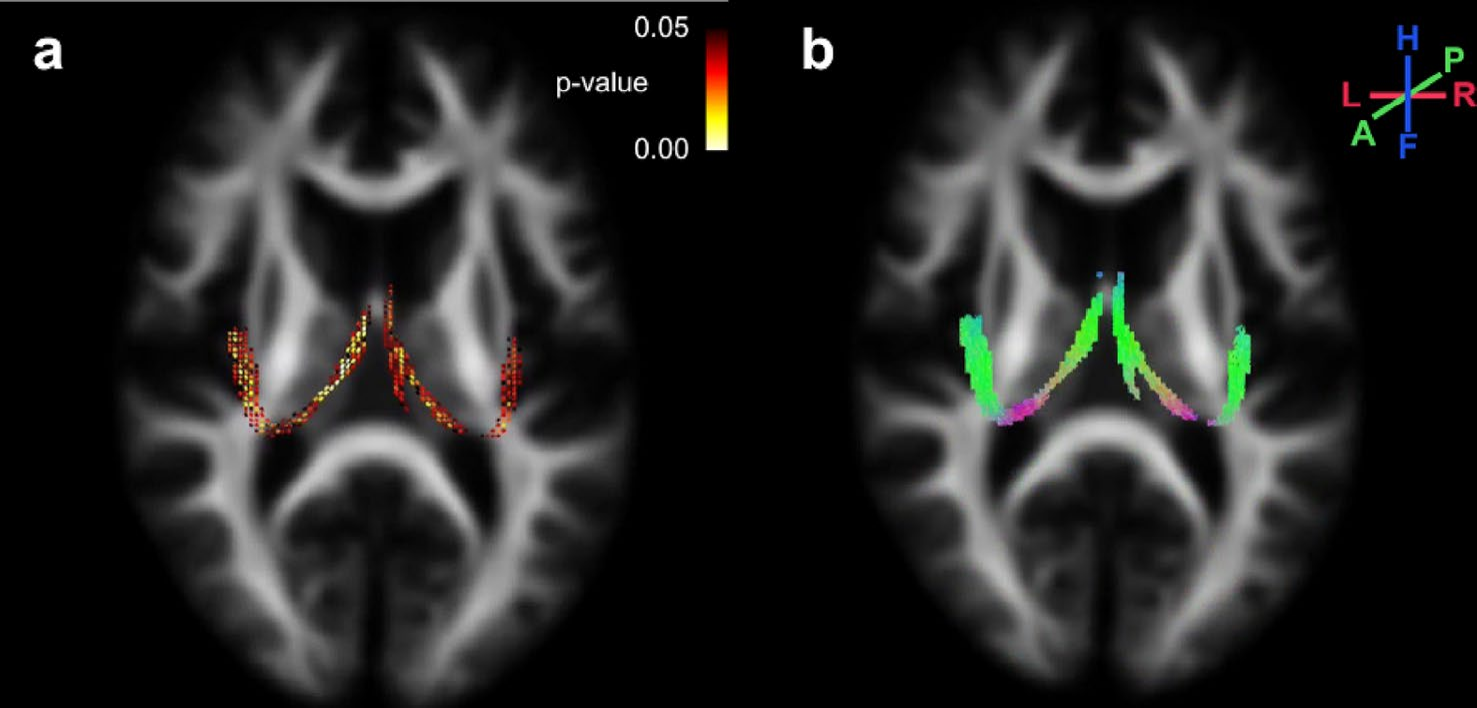
Axial images showing (a, b) white matter fixels where the FDC is significantly correlated (*p* < 0.05) with SPPB. The same significantly correlated regions are shown as reconstructed fibre tracts coloured by their primary direction: Red for left‐to‐right (L–R), green for anterior‐to‐posterior (A–P), and blue for head‐to‐foot (H–F).

Structural white matter measures, as examined using fixel‐based diffusion MRI, were associated with SPPB performance. FD and FDC in visual and thalamo‐occipital tracts, including the right optic radiation (13%), right thalamo‐occipital tract (15%) and left fornix (13%), were positively associated with higher SPPB scores (Table 4). These findings suggest a possible compensatory role of visual input in postural control for older people with pain‐related balance impairment. The associations were primarily driven by fibre density, implying that higher axonal packing within visual‐sensory tracts supports better gross motor function (Figure 1).

**TABLE 4:** White matter tracts showing significant associations with SPPB.

White matter tract	% significant fixels^a^
Thalamo‐occipital (right)	15%
Fornix (left)	13%
Optic radiation (right)	13%
Thalamo‐parietal (right)	10%
Thalamo‐prefrontal (left)	5%

^a^ Significant associations were identified using FBA. Each fixel is associated with a specific fibre bundle. Tracts are listed in descending order of proportion of significant fixels. Tracts not listed showed no significant associations with SPPB (0% fixels were significant).

Tissue conductivity analyses revealed widespread associations with physical performance metrics. Faster daily‐life walking speed was associated with higher conductivity in the right cerebellar white matter and bilateral medial orbitofrontal cortices (*p* < 0.006). Walking duration was positively associated (i.e., longer walking duration) with conductivity in the left inferior parietal cortex (*β* = +18.0 ± 7.20 min/day, *p* = 0.0189), while higher levels of total and incidental physical activity were linked to conductivity in the bilateral cerebellar cortex, right hippocampus, right inferior parietal lobe and left rostral middle frontal gyrus. Furthermore, greater planned exercise participation (hours/week) was positively associated with conductivity in the right pallidum, suggesting a role for basal ganglia structures in initiating structured activity (Table 5).

**TABLE 5:** Associations between brain region tissue conductivity and physical function.

Outcome	Region	*β* ± SD	*p*
Daily‐life walking speed (m/s)	R cerebellum WM	7.24 ± 12.9	0.0034
	L medial orbitofrontal	4.22 ± 9.93	0.0218
	R medial orbitofrontal	15.45 ± 29.4	0.0056
Walking duration	L Inferior Parietal	18.0 ± 41.3	0.0189
Total activity	R hippocampus	358.5 ± 888.6	0.0283
	L cerebellum cortex	304.2 ± 830.8	0.0450
	R cerebellum cortex	333.9 ± 742.5	0.0156
	R inferior parietal	405.5 ± 848.3	0.0107
	L rostral middle frontal	553.5 ± 1087.3	0.0070
Incidental activity	R cerebellum cortex	281.3 ± 769.1	0.0453
	R inferior parietal	358.0 ± 875.3	0.0265
	L rostral middle frontal	526.4 ± 1105.0	0.0109
Planned exercise	R pallidum	103.2 ± 250.9	0.0256

### 3.4 Associations With Executive Function

No significant associations were found between grey matter volume and executive function measures, including motor inhibition (ReacStick OFF Accuracy) and Trail Making Test (TMT) in individuals with CLBP.

**FIGURE 2:**
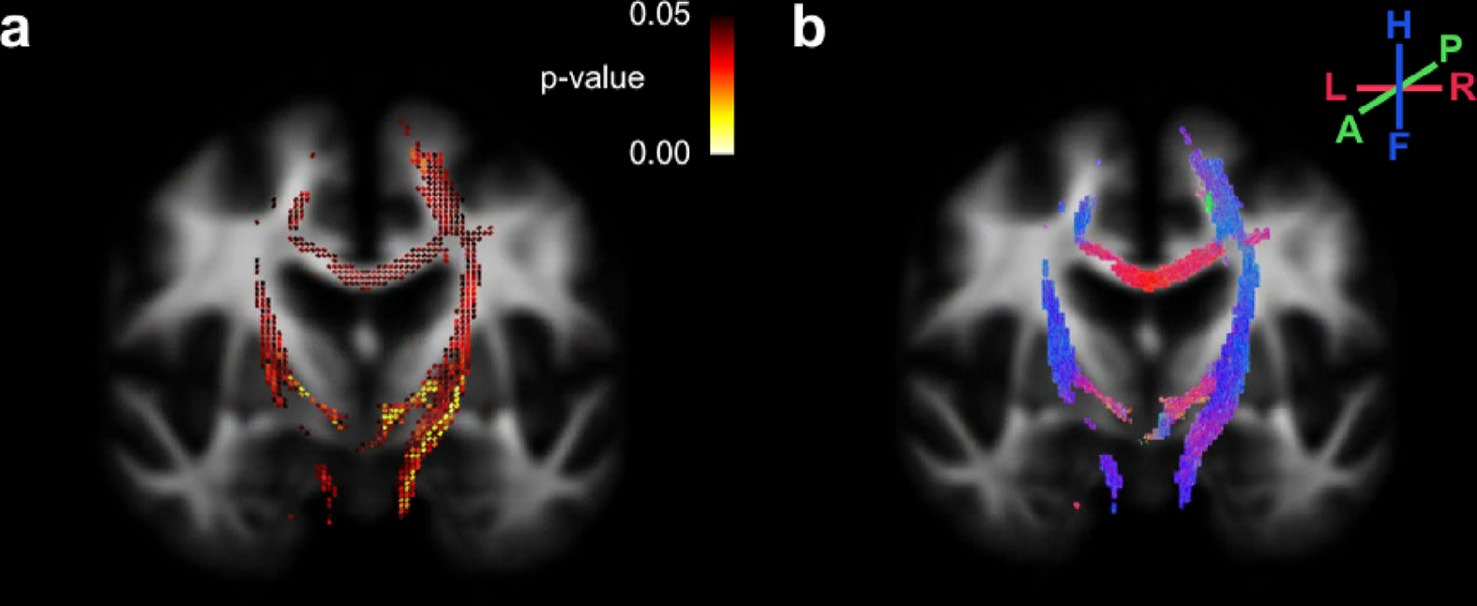
Coronal images showing (a, b) white matter fixels where the FDC is significantly correlated (*p* < 0.05) with ReacStick off accuracy. The same significantly correlated regions are shown as reconstructed fibre tracts coloured by their primary direction: Red for left‐to‐right (L–R), green for anterior‐to‐posterior (A–P), and blue for head‐to‐foot (H–F).

White matter FDC was significantly associated with motor inhibition as measured by ReacStick OFF Accuracy. Widespread associations were observed across key sensorimotor and thalamic pathways, with the highest percentage of associations in white matter occurring in the left corticospinal tract (59%), left parieto‐occipital pontine tract (47%), midbody of the corpus callosum (anterior: 41%; posterior: 42%) and superior thalamic radiation (37%) (Table 6). These associations were primarily driven by FC, indicating that larger cross‐sectional areas of major white matter bundles support faster inhibitory responses (Figure 2). The limited involvement of prefrontal white matter projections suggests that fast motor inhibition in CLBP is more dependent on subcortical and sensorimotor circuits than on higher‐order executive pathways.

**TABLE 6:** White matter tracts showing significant associations with OFF accuracy.

White matter tract	% significant fixels^a^
Corticospinal tract (left)	59%
Parieto‐occipital pontine (left)	47%
Corpus callosum—Posterior midbody	42%
Corpus callosum—Anterior midbody	41%
Corpus callosum–All	40%
Thalamo‐precentral (left)	39%
Superior thalamic radiation (left)	37%
Fronto‐pontine tract (left)	36%
Striato‐precentral (left)	34%
Striato‐postcentral (left)	22%
Thalamo‐postcentral (left)	22%
Thalamo‐prefrontal (left)	22%
Striato‐parietal (left)	19%
Superior cerebellar peduncle (left)	17%
Corticospinal tract (right)	15%
Corpus callosum–Genu	9%
Thalamo‐parietal (right)	9%

^a^ Significant associations were identified using FBA. Each fixel is associated with a specific fibre bundle. Tracts are listed in descending order of proportion of significant fixels. Tracts not listed showed no significant associations with Off‐accuracy (0% fixels were significant).

Tissue conductivity analyses revealed complementary insights into executive function. For ReacStick OFF Accuracy, higher conductivity in the left medial orbitofrontal cortex (*β* = +7.58 ± 2.84, *p* = 0.0128) was significantly associated with improved inhibitory control, pointing to a functional role of frontal lobe biophysics in executive processing (Table 7).

**TABLE 7:** Associations between brain region tissue conductivity and executive function.

Outcome	Region	*β* ± SD	*p*
OFF‐accuracy (ReactStick)	L medial orbitofrontal	7.6 ± 16.3	0.0128

## 4 Discussion

This study provides novel evidence that daily‐life gait quality, an ecologically valid marker of real‐world balance and fall risk, is associated with brain integrity in older people with CLBP. Interestingly, these associations were captured by tissue conductivity rather than conventional structural MRI, suggesting that microstructural changes in cerebellar and subcortical regions may contribute to gait impairment before overt degeneration is detectable. Gait quality was strongly associated with tissue conductivity in cerebellar white matter, pallidum and thalamus. The cerebellum's role in fine motor coordination and the thalamus's role in sensorimotor integration likely underlie these associations (Manto et al. 2012; Tyll et al. 2011). This interpretation aligns with resting‐state fMRI studies showing that stronger cerebellar‐motor connectivity relates to lower gait variability and that thalamic integrity supports gait speed and postural control in older people (Massa et al. 2019). Although the specific tissue properties reflected by the conductivity measures cannot be determined from these data, the findings suggest that preservation of biophysical properties of the cerebellum and subcortical grey matter may play an important role in supporting balance and mobility in older people with CLBP.

Physical performance showed broader neural associations across the three imaging modalities. SPPB performance was associated with higher fibre density in visual and thalamo‐occipital tracts and with larger grey matter volumes in subcortical regions, while walking duration was associated with larger grey matter volumes in subcortical and cerebellar regions. In addition, conductivity across cerebellar, basal ganglia and frontal areas was associated with faster daily‐life walking speed, longer walking duration and greater physical activity levels. Larger volumes in motor‐related subcortical regions, including the thalamus, hippocampus and putamen, were associated with higher SPPB scores, suggesting that structural preservation in these areas supports functional mobility (Gooijers et al. 2016). Specifically, hippocampal volume has been linked to navigation and spatial awareness, which may indirectly facilitate complex gait patterns such as obstacle negotiation, while putamen volume correlates with gait speed and lower‐extremity strength in older people (Beauchet et al. 2019; Nedelska et al. 2012; Pieruccini‐Faria et al. 2023). Greater thalamic, cerebellar and brainstem volumes were also associated with longer daily walking duration, whereas increased CSF (a marker of atrophy) was linked to reduced activity (De Vis et al. 2016). In contrast, SPPB performance showed only modest associations with white matter, primarily in visual and thalamo‐occipital pathways, which may indicate a greater reliance on visual input for postural control this sample, consistent with findings reported in CLBP populations which reflects a diminished proprioceptive accuracy in the trunk and lower limbs (Mohammadi et al. 2021; Sheeran et al. 2012). This agrees with findings that people with CLBP rely more on optic flow during balance tasks (Sung and Lee 2025). These findings suggest that while grey matter structure and white matter connectivity underpin core physical capacities (e.g., gait and strength), tissue conductivity may capture more dynamic aspects of neural efficiency and coordination, particularly across motor, cognitive and motivational circuits that support real‐world activity engagement in older people with chronic low back pain.

Subcortical conductivity provided further insight, and was associated with real‐world mobility in this study. Faster daily‐life walking speed was associated with greater conductivity in the cerebellum and medial orbitofrontal cortices, highlighting joint contributions of subcortical motor control and frontal executive modulation to real‐world walking (Hausdorff et al. 2008). In addition, higher conductivity in regions including the right hippocampus, bilateral cerebellar cortex, right inferior parietal lobe and left rostral middle frontal gyrus was associated with greater physical activity levels, while higher conductivity in the right pallidum was associated with planned exercise participation. These findings suggest that preserved tissue conductivity across regions involved in motor coordination, cognitive integration and motivational processes may support everyday activity engagement in older people with CLBP. The pallidum's association with planned exercise is consistent with its role in intentional motor behaviour (Calabresi et al. 2014). The hippocampus may also contribute to mobility through its involvement in navigation and spatial awareness, which may facilitate complex gait behaviours and environmental interaction (Gooijers et al. 2016). Although emotional‐motor integration and fear‐avoidance processes are recognised contributors to mobility limitations in chronic pain (Veinante et al. 2013), psychological factors were not directly assessed in this study. Given the cross‐sectional design and absence of a pain‐free comparison group, it is not possible to determine whether these associations are specific to CLBP or reflect broader ageing‐related processes. Future studies incorporating direct measures of fear‐avoidance and longitudinal assessments are needed to clarify how neural mechanisms influence activity participation in older people with chronic pain.

Executive function, as measured by OFF Accuracy, was associated with widespread white matter pathways (dominated by fibre cross‐section) and to orbitofrontal cortex conductivity, while Trail Making Test was not associated with any. These findings are consistent with prior work linking white matter integrity to both gait performance and fall risk in older adults, suggesting that structural connectivity may underpin the rapid sensorimotor processing required for balance recovery (Callisaya et al. 2014; He et al. 2021). Specifically, white matter associations spanned a broadly distributed network of sensorimotor and thalamic white matter tracts, driven primarily by fibre cross‐section. Research demonstrated that fractional anisotropy in frontal white matter projections of the corpus callosum correlates with inhibitory performance, highlighting the critical role of large‐calibre pathways in supporting rapid inhibition (Treit et al. 2014). Diffusion MRI work has also shown that higher fibre cross‐section in these tracts predicts faster stop‐signal reaction times in older adults, linking tract calibre with inhibitory control performance (Coxon et al. 2012). Orbitofrontal cortex conductivity was associated with OFF accuracy, implying that local grey matter microstructure and adjacent short‐range white matter fibres in prefrontal regions support inhibitory processing (Banich and Depue 2015). The orbitofrontal cortex is a key region involved in inhibitory control and behavioural regulation, and its involvement further supports the link between executive processes and mobility outcomes (Brockett and Roesch 2021). This aligns with studies showing reduced orbitofrontal connectivity predicts slower inhibitory control (Bryden and Roesch 2015). Interestingly, poor inhibitory control has been suggested to precede chronic pain in older adults, with deficits in executive function associated with a higher risk of developing pain over time (Rouch et al. 2021). This aligns with our finding of relatively low OFF accuracy, highlighting a potential avenue for future investigation. It is noteworthy that Trail Making Test performance did not show significant associations with white matter or conductivity metrics in this sample. One possible explanation is that OFF Accuracy taps into rapid motor inhibition (~390 ms), which relies more heavily on subcortical and sensorimotor circuits, whereas TMT involves slower, sequential inhibition and may engage more distributed pathways (Fellows et al. 2017; Ray Li et al. 2008). The homogeneity of this healthy, active sample may have further limited TMT variability. In addition, mechanistically, inhibitory control plays a critical role in rapidly suppressing inappropriate motor responses and enabling adaptive reactions to environmental perturbations. As proposed by Bolton and Richardson (2022), perceptual inhibition allows rapid prioritisation of relevant sensory input during balance challenges, while motor inhibition enables timely interruption of ongoing movements (e.g., walking) when stability is threatened. Taken together, these findings suggest that both white matter microstructure and orbitofrontal cortical properties may contribute to inhibitory control processes that are critical for maintaining balance and preventing falls, particularly in situations requiring rapid motor adjustments.

The multimodal imaging approach revealed distinct but complementary patterns. FBA highlighted the role of fibre density in visual‐sensory tracts for SPPB and of fibre cross‐section in sensorimotor pathways for rapid inhibitory control. Volumetric grey matter measures identified subcortical and cerebellar regions whose preservation supports general mobility and walking activity (Soulard et al. 2020). While no prior study has directly linked EPT‐derived brain conductivity to gait quality and mobility, prior work shows that MRI‐based conductivity maps can detect neural activity changes. For example, Cao et al. (2024) used phase‐sensitive MRI methods to map conductivity changes associated with neural activity in the motor cortex, illustrating EPT's sensitivity to microstructural alterations that underlie motor function. Taken together, these modalities collectively indicate that different aspects of brain structure and microstructure may be associated with macrostructural white matter integrity which facilitates fast motor responses, volumetric grey matter preservation which supports broader physical function, and microstructural tissue health which underlies nuanced real‐world balance, cognitive function and daily activity.

Several limitations must be acknowledged. First, the sample size was modest, and multiple comparisons increase false positives; findings should therefore be interpreted as preliminary and hypothesis‐generating rather than definitive. Second, the cross‐sectional design prevents causal inferences; longitudinal studies are needed to determine whether changes in brain integrity precede or follow functional decline. Third, the absence of a pain‐free control group limits our ability to disentangle CLBP‐specific effects from normal ageing. In addition, history of falls was not directly assessed in this study, limiting our ability to relate brain measures to past fall events. However, the primary outcome of daily‐life gait quality has been previously shown to be a strong predictor of future falls, supporting its use as a proxy measure of fall risk. Fourth, participants were relatively healthy, physically active (walking ~1 h/day), highly educated, and predominantly female, with only 18% reporting severe pain, which may limit generalisability. It remains unclear whether similar brain–function associations would be observed in less active or more severely affected populations, as suggested by our proposed pain–fall risk pathways (Seydi et al. unpublished manuscript). Finally, although we adjusted for age, sex, education and intracranial volume, residual confounding by factors such as medication use, comorbidities and pain duration is also possible. In addition, pain intensity and duration were not included as covariates in the regression models, which may have influenced the observed associations. Future work should replicate these findings in larger, more diverse cohorts, include pain‐free controls, and employ longitudinal designs to clarify whether changes in conductivity and white matter integrity precede functional decline or reflect compensatory processes.

### 4.1 Clinical Implications

Our findings support expanding CLBP management beyond musculoskeletal rehabilitation to include neural and cognitive targets (Brumagne et al. 2019). First, the observed associations between cerebellar and subcortical conductivity and gait quality suggest that these regions may represent candidate neural markers of balance and fall risk, although this requires validation in larger and controlled studies. Second, the involvement of cerebellar and thalamic regions supports existing evidence for the importance of balance and coordination training but does not establish causality. Third, the association between executive function, inhibitory control, and both white matter tract calibre and orbitofrontal conductivity suggests that cognitive processes may contribute to mobility outcomes, supporting further investigation of cognitive‐motor training into rehabilitation (Schoene et al. 2014). Finally, the relationship between hippocampal conductivity and physical activity measures suggests that limbic regions may be involved in behavioural aspects of movement in individuals with CLBP, although psychological factors such as fear‐avoidance were not directly assessed in this study and require further investigation (Vergeld et al. 2021). If replicated, these findings suggest that brain integrity in cerebellar and subcortical regions contributes to real‐world mobility in older people with CLBP, supporting the rationale for interventions that target both neural and motor function to reduce fall risk.

## 5 Conclusion

In older people with CLBP, daily‐life gait quality was associated with tissue conductivity in cerebellar and subcortical regions, in the absence of associations with conventional structural MRI measures. Executive function relied on widespread white matter pathways and orbitofrontal conductivity, while physical function reflected a combined contribution of visual‐sensory white matter, subcortical and cerebellar volume, and conductivity in motor‐related circuits. These findings emphasise the importance of cerebellar, subcortical and sensorimotor networks in fall risk and mobility. Larger longitudinal and intervention studies are needed to determine whether targeting these neural substrates can preserve balance and quality of life.

## Author Contributions

M.S. conceived the study, collected and analysed the data, developed the methodology, prepared the visualisations, and drafted and revised the manuscript. K.D. provided supervision, contributed to study conceptualisation and methodology, oversaw project administration, and critically reviewed and edited the manuscript. M.A. contributed to manuscript review and editing. J.K.R. assisted with data analysis, interpretation and manuscript review. C.D.R., J.C., P.S.S., L.L. and M.A.G. contributed to the neuroimaging methodology, data validation, analysis, and manuscript review. K.S.S. provided supervision, contributed to conceptualisation, methodology development, data analysis, and project administration, and critically reviewed and edited the manuscript.

## Funding

James Richardson's academic work is supported by the Newman Family Foundation Gift. Additional support was provided by the ARC Research Hub for Connected Sensors for Health. This research was also supported by the UIPA PhD scholarship from the UNSW and the Pearl PhD program from NeuRA. Kim Delbaere is supported by an NHMRC Investigator Grant (APP1193766). Louise Lavrencic is supported by a Dementia Australia Research Foundation Post‐Doctoral Fellowship.

## Conflicts of Interest

Dr. James Richardson is a co‐developer of the ReacStick device and was an author of the original validation study of the recognition reaction time test (RTclin). While the ReacStick was used in this study, Dr. Richardson did not receive any financial compensation related to its use in this research. All other authors declare no competing interests. P.S.S. reports advisory engagement with Biogen Australia (2020–2021), Roche Australia (2022), Eli Lilly (2025) and Novo Nordisk (2025), unrelated to this research.

## Supporting information


**Data S1:** STROBE Statement—Checklist of items that should be included in reports of cross‐sectional studies.
